# Case of the Simultaneous Existence of Eight Tape-Worms in the Same Individual

**Published:** 1815-01

**Authors:** M. Broussais

**Affiliations:** First Physician to the Army in Spain.


*For the Medical and Physical Journal.*

*Case of the simultaneous Existence of Eight Tape-Worms in the same Individual;* by M. BROUSSAIS, First Physician to the Army in Spain.

A SERJEANT was received into the Military Hospital in the month of November, 1813. He had been attacked with a tertian fever, which had already relapsed several times. He was pale, and slightly anasarcous. His appetite was good; but he had diarrhœa, and several times voided considerable portions of tape-worm.

As bark internally administered disagreed with him, Mr. Martel, under whose care he then was, undertook to destroy the fever by the sole employment of frictions, with alcoholic tincture. This remedy, which I have myself often employed with success in cases where the internal use of bark could not be borne, speedily cured the fever.

Notwithstanding the patient was freed from the fever, he remained weak and anasarcous, with cough, and oppression about his chest. He complained of a sense of painful weakness in the inward parts of the abdomen, which he ascribed to the worm.

At the end of five or six days, he suddenly complained of an insupportable coldness, which nothing could abate, and which threatened a return of the fever; but, instead of the hot fit, an œdematous and painful swelling of the right arm suddenly arose, the swelling of which increased to the greatest possible possible degree in the course of the night. In the morning, we found the patient trembling, his respiration laborious, with the expression of death in his countenance. He expired in the course of the day.

The body was opened. The lungs were free from adhesions, but they were filled with a sero-mucous liquid. In the abdomen, a quantity of yellow serum was found. The mucous membrane of the intestines was diseased throughout its whole extent, appeared of a light red colour, and in some isolated spots disorganized and thickened. In the jejunum and ilium we discovered eight tæniæ. They were separated, and at some distance from each other. One alone was found in the colon. Each was curled up into a ball. We measured the only three that could be disentangled. They were from ten to fourteen feet in length, and were dead.

